# Emotionality Stigma, Sociocultural Factors, and Health Inequities in Urban Adolescents

**DOI:** 10.3390/ijerph22101500

**Published:** 2025-09-30

**Authors:** Hayley D. Seely, Eileen Chen

**Affiliations:** 1 Department of Human Development & Family Studies, Colorado State University, Fort Collins, CO 80523, USA; 2 Denver Health and Hospital Authority, Denver, CO 80204, USA

**Keywords:** adolescent mental health, adolescent substance misuse, urban populations, emotionality stigma, health inequity

## Abstract

Stigmatized views of emotionality form within familial, cultural, and societal contexts and serve as a mechanism impacting youth mental health and substance misuse with notable ties to health equity. Yet critical questions remain regarding the impact of racial identity on emotionality stigma in urban groups and the moderating relationship between race and emotionality stigma on youth mental health and substance misuse. The current study aimed to investigate emotionality stigma as a mechanism of health inequity by exploring the relationships between racial identity, emotionality stigma, and adolescent mental health and substance misuse. Urban adolescents (*n* = 85) recruited from a combined mental health and substance use treatment program reported on their stigmatized views of emotionality, mental health, and substance use. Participants primarily identified as multicultural (60.3%) and socioeconomically disadvantaged, with 55.2% requiring transportation assistance and 63.8% being either insured through Medicaid or uninsured. Findings suggest a link between racial identity and emotionality stigma that was associated with attachment (*β* = −3.43, *p* < 0.001) as well as substance misuse type (*β* = 5.36, *p* < 0.001) and polysubstance use (*β* = −6.53, *p* < 0.001) for urban adolescents in combined treatment. This study is the first to provide empirical support for the interconnected role of sociocultural factors and emotionality stigma and calls for systems-level change to address emotionality stigma individually, communally, and socially.

## 1. Introduction

Mental health and substance misuse concerns are highly comorbid in adolescent populations, greatly impacting social, emotional, and academic functioning, leading to poorer treatment outcomes, and increasing risk of continued symptomology throughout life [1,2,3,4,5,6,7,8]. Although this comorbidity and the associated impacts are well-documented, few integrated treatment options exist and access to care can be a substantial barrier contributing to continually rising prevalence rates [9,10]. Thus, increasing understanding of the contributors to comorbid mental health and substance misuse is an essential step for research toward improving and expanding practice.

Although mental health and substance misuse concerns have wide-spread impact, youth living in urban environments are at disproportionate risk [11]. Given the unique environmental stressors including exposure to community violence, inequity, resource and economic insecurity, and population density, mental health concerns are even more common in urban youth [11]. This increased prevalence further perpetuates rates of substance misuse [12], and worse, accidental death [13]. Despite the clear need for treatment options specific to the unique stressors and needs of urban youth, current treatment options rarely consider these stressors and are often difficult to access for those who need them most due to structural inequities [14,15].

To address this gap, our team [16] developed an intensive outpatient program (IOP) for treating co-occurring mental health and substance misuse concerns amongst adolescents in an urban metropolitan area. This IOP was intentionally developed centering sociocultural factors and embedding interventions to increase access to care. Key components of this IOP include the integration of contextually fluid therapeutic techniques, leveraging emotionality stigma theory [17,18], and introducing barrier reduction techniques. Following the positive outcomes of this program [16], including adolescent achievement of negative urine drug screens from all substances (43.4%) and primary substance (69.7%), similar approaches were embedded within the traditional outpatient program of the clinic as well. Despite the preliminary success, it is important to further investigate specific components of this program and how they impact mental health and substance misuse in urban adolescent populations. Because difficulties tolerating distress and regulating emotions are known risk factors for substance use [19,20], it is critical to consider how these mechanisms interact with sociocultural processes. In particular, examining these dynamics through the lens of emotionality stigma offers a novel direction for research. Thus, while there is research to support the continued use of therapeutic technique integration and barrier reduction techniques, emotionality stigma is a novel component of this program that needs further exploration.

Emotionality stigma theory posits that emotions become stigmatized through the emotion socialization process such that youth learn that some emotional experiences and expressions are acceptable, while other experiences and expressions are stigmatized [17,18]. The resulting stigma formed through the socialization process is dependent on sociocultural factors as the emotion socialization process is dictated by cultural norms and family values [21,22]. In other words, families, communities, and society more generally influence youths’ perceptions of which emotions are acceptable for whom and in what contexts. Although emotionality stigma is a relatively new construct, the idea that youths’ emotion management is influenced by others has a long history. Indeed, emotion socialization research more generally illustrates a direct connection between parental socialization practices and youth emotion management [23]. This process is influenced both by familial and cultural norms [24] and developmental stages with adolescence serving as is a pivotal time for emotion regulation development specifically [25]. Extensive research has also investigated the impact of feeling emotions incongruent with the context and the effort it takes to purposefully influence one’s own emotions toward increased congruence (emotion work) [26,27,28]. This research has highlighted the social norms surrounding emotionality and how this impacts youth more generally.

Grounded in emotion socialization and stigma research, emotionality stigma specifically has been found to be related to poorer mental health outcomes in youth [17] and has been recognized as a potentially impactful variable in the intergenerational transmission of mental health [18]. Indeed, preliminary research suggests that a parent’s emotion expression may influence youth’s mental health through their endorsement of emotionality stigma [18]. However, research has yet to explore (1) how sociocultural factors impact the development or endorsement of emotionality stigma, (2) the extent to which emotionality stigma is related to both mental health and substance misuse in urban youth, and (3) the interacting role emotionality stigma and sociocultural factors play in youth mental health and substance misuse.

The current study addresses these gaps in current research by examining these relationships in a sample of youth engaging in co-occurring substance use and mental health treatment in an urban outpatient setting. The current study had three main aims:

Aim 1. Explore the role of sociocultural factors, including race and socioeconomic status, in adolescent substance misuse and emotionality stigma endorsement.

Aim 2. Examine the relationship between emotionality stigma, mental health, and substance misuse in urban youth.

Aim 3. Investigate race as a moderator in the relationship between emotionality stigma and mental health and substance misuse.

Though this study is exploratory in nature, based on past and preliminary research [11,12,13,14,15,16,17], we expected urban youth with elevated sociocultural risk factors to be at increased risk of polysubstance use, use of substances with high misuse potential (e.g., cocaine, fentanyl, methamphetamines), and emotionality stigma endorsement. We also hypothesized, based on emotionality stigma theory and past research with adolescent populations [17], that emotionality stigma would be related to mental health and substance misuse concerns. Finally, given the differences in emotion socialization often observed based on racial identity [17,18,21], we anticipated that race would moderate the relationship between emotionality stigma and urban youth outcomes, though the specifics of this moderation were exploratory. Overall, through the findings of this study, we aimed to advance emotionality stigma research in urban adolescent populations and inform future clinical work utilizing the emotionality stigma framework.

## 2. Materials and Methods

The data utilized in this manuscript are a part of routine outcomes monitoring in an urban adolescent treatment setting for comorbid mental health and substance misuse concerns. Youth who meet criteria for a 2.1 level of clinical care, defined by the American Society of Addiction Medicine (ASAM) based on six dimensions (e.g., Acute Intoxication and/or Withdrawal Potential; Biomedical Conditions and Complications; Emotional, Behavioral, or Cognitive Conditions and Complications; Readiness to Change; Relapse, Continued Use, or Continued Problem Potential; and Recovering/Living Environment [29]) were enrolled into traditional outpatient therapy and/or intensive outpatient therapy. Youth needing this level of care report on their mental health and emotionality stigma endorsement throughout the course of treatment to inform treatment targets and progress. These surveys are completed with the youth’s individual or group therapist to allow for treatment tracking. However, the data is anonymized for research purposes; youth are provided with unique codes for the surveys, and no identifying information is recorded or saved. This routine outcomes monitoring and its use for publication have been reviewed and approved for exemption by the Colorado Multiple Institutional Review Board (COMIRB #25-1475).

The targeted sample size was 92 youth (50% youth of color) based on an a priori power analysis using G*Power (version 3.1, Heinrich Heine University Düsseldorf, Düsseldorf, Germany) [30], with an alpha of 0.05, power of 0.80, and an estimated effect size of 0.15. All patients (*N* = 170) who qualified for ASAM Level 2.1 and were seen for treatment between January 2022 and August 2025 were asked to complete outcomes monitoring. Youth (*M_age_* = 16.06, *SD_age_* = 1.45) self-identified their gender, race, and transportation needs. Other demographic variables including insurance coverage and primary substance were extracted from the youths’ medical charts. For sample demographics, see Table 1.

The routine monitoring survey completed by adolescents included the Emotionality Stigma Scale—Adolescents (ESS-A) [17] and two measures of mental health: (1) the Valuing Questionnaire (VQ) [31] and (2) the Adolescent Attachment (AAQ) [32]. The ESS-A is a 16-item measure that was adapted from the Emotionality Stigma Scale (ESS) [33] to assess emotionality stigma in adolescent populations. The ESS-A has illustrated validity and reliability evidence [17]. This scale includes three subscales: stigma endorsement, stigma resistance, and differential treatment. Urban youth utilized a 4-point Likert Scale to report their agreement on each item (1= strongly disagree; 4= strongly agree). Items associated with the stigma resistance subscale were reverse coded. Subscale mean scores were then calculated with higher scores indicating higher stigma endorsement, less stigma resistance, and more reported differential treatment, respectively. All ESS-A items were averaged to create a total score as well with higher values indicating more emotionality stigma overall (α = 0.782). The VQ is a self-report scale designed to measure the extent to which an individual is living in line with their values. Across 10 items, urban youth indicated their values alignment using a 7-point Likert Scale (0 = not true at all; 6 = completely true). Negatively worded items were reverse coded. A mean score was utilized to account for missing data, with higher scores indicating more alignment with values. The VQ illustrated adequate reliability in our sample with an internal consistency of α = 0.863. Lastly, as another measure of the youth’s mental health, the AAQ was included to assess the youth’s attachment to their primary caregiver. Urban adolescents reported on their level of agreement with the 9 items using a 5-point Likert Scale (1 = strongly disagree; 5 = strongly agree). Following reverse coding for negatively worded items, a mean score was created (α = 0.884).

To address our hypotheses, we conducted a series of regression analyses in IBM SPSS (version 31, Chicago, IL, USA). For these analyses, categorical variables were dichotomized to increase sample size; continuous variables remained continuous. First, we investigated the role of sociocultural factors in substance misuse and treatment dropout utilizing our full sample. Specifically, using separate analyses for each outcome, substance use type, polysubstance use, and treatment dropout were regressed on age, gender, race, insurance, and transportation. Similarly, with the sample who engaged in treatment, and thus participated in routine outcomes monitoring, we conducted regression analyses with the included sociocultural factors predicting emotionality stigma overall, as well as each dimension of emotionality stigma individually. Second, we assessed differences in mental health and substance misuse based on emotionality stigma, again using regression analyses in SPSS. Finally, we added race as a moderator in the relationship between emotionality stigma and urban youth mental health and substance misuse. For these final regression analyses, race, emotionality stigma, and an interaction variable (race × emotionality stigma) were used as predictors of youth mental health and substance misuse. Significant moderation results were probed by repeating the analyses individually for each race. The assumptions of linear regression were met within the current dataset and there was no evidence of multicollinearity (Table 1).

## 3. Results

Prior to hypothesis testing, descriptive statistics were conducted to better understand the sample and to assess for missing data. Given the known barriers to adolescent substance use treatment, it was not surprising that out of 170 patients who were referred for care and qualified for ASAM 2.1 at intake, only 85 engaged in treatment (58.8% youth of color). Using Little’s Missing Completely at Random test [34], data was determined to be missing at random, χ^2^(158) = 181.04, *p* = 0.101. Though treatment dropout is common, especially for traditionally underserved youth [35], we added treatment dropout into our analyses to assess differences in dropout based on sociocultural factors.

With the full sample (*N* = 170), we investigated substance use and dropout differences based on sociocultural and ecological variables. No differences emerged based on gender, race, nor insurance coverage suggesting substance use and dropout in our sample were unrelated to these sociocultural factors. However, older adolescents reported more polysubstance use (*β* = 0.22, *p* = 0.012, *95% CI* = 0.02–0.14) and use of other substances with high misuse potential (*β* = 0.18, *p* = 0.046, *95% CI* = 0.001–0.12). Additionally, transportation needs were associated with polysubstance use (*β* = 0.18, *p* = 0.036, *95% CI* = 0.01–0.35) and use of other substances with high misuse potential (*β* = 0.21, *p* = 0.018, *95% CI* = 0.04–0.37).

With the data from the 85 patients who engaged in treatment and participated in the outcomes monitoring, we addressed our second two aims by investigating sociocultural differences in emotionality stigma and the role emotionality stigma plays in substance use and mental health. Due to sample size restrictions, for the purpose of analyses, race, substance use type, and polysubstance use were transformed into dichotomous variables. The dichotomous variable coding is as follows: race (1 = youth of color, 2 = White youth); substance use type (1 = cannabis or alcohol use, 2 = use of other substances with high misuse potential); polysubstance use (0 = monosubstance use, 1 = polysubstance use). Bivariate correlations between major study variables are displayed in Table 2.

Overall, the results illustrated few differences in emotionality stigma based on sociocultural factors. Specifically, youth reported similar levels of total emotionality stigma across different age, sex, and socioeconomic groups. However, youth of color reported less total emotionality stigma than their White peers (*β* = −0.27, *p* = 0.037, *95% CI* = −0.41–−0.01). Furthermore, when investigating differences in the three domains of emotionality stigma, those with private insurance reported higher emotionality stigma endorsement (*β* = 0.27, *p* = 0.044, *95% CI* = 0.01–0.71) than their peers with Medicaid or who were uninsured. Compared to boys, girls simultaneously reported more stigma resistance (*β* = −0.28, *p* = 0.023, *95% CI* = −0.81–−0.06) and more perceived societal stigma (*β* = 0.39, *p* < 0.001, *95% CI* = 0.24–0.87). Consistent with less emotionality stigma overall, youth of color endorsed less perceived societal stigma (*β* = −0.24, *p* = 0.041, *95% CI* = −0.63–−0.01).

The final aim of the current study was to explore the interaction between emotionality stigma and race on mental health and substance misuse. Given that our small sample was divided into two groups (youth of color *n* = 50; White youth *n =* 35) for the purpose of these moderation analyses, the following results should be interpreted with caution. An emotionality stigma by race interaction emerged for type of substance misuse (*β* = 5.36, *p* < 0.001, *95% CI* = 1.31–2.21) and polysubstance use (*β* = −6.53, *p* < 0.001, *95% CI* = −2.51–−1.85). When probing these analyses, the effect of emotionality stigma on type of substance misuse became non-significant; however, the effect on polysubstance use remained significant for White youth (*β* = −0.37, *p* = 0.04, *95% CI* = −0.91–−0.02) such that higher reports of emotionality stigma was associated with monosubstance use instead of polysubstance use. Regarding mental health outcomes, emotionality stigma was related to less values aligned living (*β* = −0.59, *p* < 0.001, *95% CI* = −2.25–−1.12) and more impaired attachment (*β* = −0.47, *p* < 0.001, *95% CI* = −1.52–0.55). While the emotionality stigma by race interaction did not significantly relate to values, there was a significant interaction effect on attachment (*β* = −3.43, *p* < 0.001, *95% CI* = −2.88–−1.17) with emotionality stigma relating to poor attachment for youth of color (*β* = −0.41, *p* = 0.007, *95% CI* = −1.70–−0.29) and White youth (*β* = −0.48, *p* = 0.007, *95% CI* = −1.70–−0.29).

## 4. Discussion

The purpose of this study was three-fold: (1) to begin investigating the role of sociocultural factors in substance misuse and the endorsement of emotionality stigma, (2) to bolster findings on the relationship between emotionality stigma and mental health and substance misuse, and (3) to explore how emotionality stigma and sociocultural factors interact and inform youth mental health and substance misuse. In addition, we explored sociocultural differences in treatment dropout and found no significant differences in dropout based on race nor socioeconomic status (insurance and transportation needs). In line with past research [16], this finding may suggest that the traditional outpatient program and intensive outpatient program where youth in this study received treatment is successfully reducing known barriers to treatment that have created well documented treatment disparities [35,36]. However, additional research is needed to better understand what factors are impacting dropout for youth in the included clinic and other clinics.

In regard to our specific aims and hypotheses, the investigation of sociocultural differences in substance misuse suggested that older adolescents and those in need of transportation assistance in the included clinic had a higher likelihood of using other substances with high misuse potential, such as opioids, and engaging in polysubstance use. These findings suggest that additional support may be indicated for these two higher-risk groups. One potential support system to integrate into future treatment is a peer support network. Peer support has been identified as a highly impactful resource in substance use treatment [37] and has recently been integrated into care for youth [38]. This additional support may be uniquely beneficial for older teens and those who need transportation assistance because peer support groups foster healthy relationship building, can incorporate transitionary life skills, and often meet within the community.

Our results are also the first to empirically investigate the role of numerous sociocultural factors on emotionality stigma. Based on past research and emotionality stigma theory [17], relationships between emotionality stigma, sex, and race were expected. In line with past research [17], we found evidence of sex differences in emotionality stigma such that girls reported more stigma resistance and more perceived differential treatment based on emotionality. However, findings also showed that youth of color and those with public insurance (Medicaid) reported less emotionality stigma than White youth and those with private insurance. In other words, those with higher sociocultural risk factors seem to be at less risk of emotionality stigma than their peers with less sociocultural risk. This finding was contradictory to what was expected based on theory and past research [17] but may suggest a level of resilience in high-risk urban youth.

There are many possible reasons for these findings. First, given the historic and ongoing oppression embedded in the current social construct of race, youth of color face stigmatization that their White peers are not exposed to [39,40,41]. As a result, youth of color may be more attuned to the structural or systemic roots of emotionality stigma, leading them to differentiate between oppression and societal stigma and personal beliefs about emotional expression specifically. This explanation is supported by the fact that youth of color indeed reported less perceived societal stigma of emotionality than did their White peers. To explore the role of racial discrimination and oppression in an individual’s endorsement of emotionality stigma, future research should investigate these two variables in unison to see if endorsement of emotionality stigma in fact decreases as reports of more severe stigmatization based on race increase.

Alternatively, the shared experience of marginalization amongst youth of color may create space for emotional connection and mutual support, particularly in the treatment context, thereby reducing emotionality stigma. Compounding this, while all youth are socialized regarding the acceptability of emotionality for whom and in what context [18], this socialization process is often embedded with safety for youth of color [21] providing reasoning for regulating emotions in certain contexts. Thus, this finding may reflect the more context-dependent nature of emotional expression among youth of color compared to their White counterparts. Additionally, as the majority of youth of color in our sample identified as Hispanic or Latinx, this finding could speak to differences between White and Hispanic/Latinx youth specifically. Indeed, Hispanic/Latinx culture is often characterized by more supportive reactions and open emotion expressions compared to other oppressed groups [42,43], which would, theoretically lead to less emotionality stigma overall [17]. Thus, the lower emotionality stigma in youth of color found within our study may be reflective of Hispanic and Latinx cultural values around emotion. Finally, as all youth in our sample are considered high-risk, it is possible that White youth in our sample perceive more emotionality stigma due to embedded health inequity beliefs. In other words, these youth may perceive a mismatch between their lived experiences and dominant cultural norms and, thus, experience increased stigma. This may be especially relevant for White youth navigating social or economic hardship, whose struggles may contradict broader cultural expectations tied to race, privilege, and success. A large-scale socialization study focused on the influence of race and ethnicity in emotionality stigma for groups across different socioeconomic levels is needed to begin uncovering the nuances of emotionality stigma within context.

To address the final aim of this study, we investigated the interaction between emotionality stigma and race on youth mental health and substance misuse. Overall, results were consistent with our hypotheses and past research [17], providing partial support for the role of emotionality stigma in mental health and substance misuse, with preliminary distinctions based on sociocultural factors. As expected, emotionality stigma was associated with less values aligned living and worse attachment in our sample. Although an interaction emerged on attachment specifically, potentially illustrating differences between youth of color and White youth, the directionality remained the same such that for both youth of color and White youth, higher levels of emotionality stigma was related to poorer attachment. Interestingly, with substance use, we found that higher reports of emotionality stigma were related to monosubstance use for White youth only. Again, this could have to do with internalized societal norms related to the dominant culture such that for White youth, their experience of stigma encourages smaller deviations from what is socially expected. However, as detailed above, more research is needed to better understand the relationship between sociocultural factors, emotionality stigma, and youth substance misuse.

In addition to the proposed avenues for future work, our findings have meaningful applications to both current understanding of urban youth substance misuse and mental health and to clinical work. Most importantly, the results presented herein contribute to dismantling harmful stereotypes that equate substance misuse risk with racial identity. Our results suggest that sociodemographic and structural factors (e.g., transportation access and age) play a more meaningful role in substance misuse risk than race itself. Further, our findings suggest that the relationship between emotionality stigma and substance misuse and mental health outcomes between racial groups may be more nuanced than previously expected. Thus, framing substance use through a sociocultural and systemic lens may serve to reduce stigma and shift the narrative from one of individual blame or racialized assumptions to one that recognizes the role of context, particularly with regard to access, developmental level, and support.

Additionally, our findings highlight the potential benefit of attending to emotionality stigma in treatment, particularly as it relates to adolescent attachment and values-aligned living. In line with the well-researched multicultural orientation framework [44] which provides a guidepost for clinicians to use to foster cultural humility, cultural opportunities, and cultural comfort, providers should be aware of how internalized cultural norms around emotional suppression and expression may manifest differently across racial groups. Integrating psychoeducation and open discussion around emotionality and its interaction with cultural norms may help reduce shame and improve long-term outcomes.

Despite the important findings and application to research and practice, our study is not without limitations. Although recruiting a diverse sample of 85 youth in co-occurring mental health and substance use treatment is a strength of this study, non-clinical and larger sample sizes would be needed to investigate the intricacies of sociocultural factors, emotionality stigma, and youth mental health and substance misuse. Indeed, our small sample size increases the likelihood of Type I and II errors. Furthermore, as all participants were also recruited from a treatment-seeking population, the generalizability of our findings is limited to clinical samples. Thus, the results of this study should be understood as preliminary and thus interpreted with caution. Additionally, a replication study with a large, non-clinical sample is recommended. Furthermore, given our small sample size, we dichotomized race/ethnicity to increase our power to detect effects. While this allowed us to conduct this preliminary work, dichotomizing race/ethnicity is an oversimplification and ignores important intra-group differences. Relatedly, the majority of youth of color in our sample identified as Hispanic or Latinx. Taken together, a larger sample with more representation of other racial/ethnic backgrounds and from non-clinical settings, such as schools and community youth organizations, is an important next step. Additionally, while the integration of sociocultural factors offers a more nuanced understanding of how stigma functions across subpopulations, questions remain regarding the interaction between emotionality stigma and other sociocultural factors on youth mental health and substance use. One key and related next step is to investigate youth subpopulations based on sociocultural factors and presenting issues. It may be that there are nuanced differences in treatment dropout and outcomes based on youth sociocultural context. This would need to be investigated using a person-centered approach and could have notable ramifications for future research and treatment approaches. Finally, although we make clinical suggestions based on past research and our findings, clinical research is needed to investigate tailored treatment options to provide additional levels of support to populations with heightened risk factors.

## 5. Conclusions

Our findings suggest that sociodemographic and contextual variables, such as race, age, and transportation access, may influence how urban youth experience and respond to emotionality stigma, with possible implications for both mental health and substance use. Specifically, these exploratory analyses suggested older adolescents and those in need of transportation assistance may be at higher risk of substance misuse and girls, White youth, and those with private insurance may be at higher risk of emotionality stigma. Our findings also bolstered past research, illustrating emotionality stigma as a potential risk factor for poorer mental health for all youth, though White youth appeared to be more affected. Overall, based on this and past research [14,15,16,45,46,47], additional efforts to create accessible and tailored treatment for urban youth presenting with comorbid mental health and substance misuse concerns are warranted. The first step toward this type of care is additional research to better understand specific subpopulation needs and address the limitations of this study which include a small sample size, the dichotomization of race/ethnicity, and a treatment-seeking population. Efforts to increase access to quality care is a meaningful approach in the meantime. This includes leveraging past research that has identified barrier reduction techniques including providing transportation assistance, contingency management, meal incentives, etc. [16]. Additionally, community and social efforts to decrease stigmatization of emotionality and increase help-seeking more generally may be helpful in improving equity in behavioral health care. While further research is needed to deepen our understanding of how emotionality stigma interacts with youth identity and risk, dismantling emotionality stigma, particularly within the context of marginalized and underserved youth populations, may be an important pathway toward improving engagement, reducing risk, and fostering long-term healing.

## Figures and Tables

**Table 1:** Demographics.

Demographic Variable	*M* (*SD*)	*n* (%)
Age	16.06 (1.45)	
Gender		
Girl		51 (30.2%)
Boy		111 (65.7%)
Transgender Girl		1 (0.5%)
Transgender Boy		2 (1.2%)
Nonbinary		4 (2.4%)
Race/Ethnicity		
Hispanic		67 (39.4%)
Black/African American		10 (5.9%)
American Indian/Alaska Native		2 (1.2%)
Multicultural		13 (7.6%)
White		78 (45.9%)
Insurance		
Medicaid/Uninsured		107 (63.3%)
Private Insurance		62 (36.7%)
Transportation Needs		77 (55.4%)
Primary Substance Type		
Cannabis use		80 (47.9%)
Alcohol use		26 (15.6%)
Opioid use		50 (29.9%)
Amphetamine use		11 (6.6%)
Polysubstance Use		96 (57.5%)
Engaged in Treatment		85 (50.0%)

**Table 2:** Bivariate Correlations and Descriptives of Major Study Variables.

Measure	1	2	3	4	5	6
1. Emotionality Stigma	__					
2. Race	0.34 **	__				
3. Substance Use Type	−0.14	−0.01	__			
4. Polysubstance Use	−0.22	0.02	0.35 ***	__		
5. Values Alignment	−0.62 ***	−0.28 *	0.16	0.20	__	
6. Attachment	−0.49 ***	−0.21	0.23 *	0.18	0.60 ***	__
Mean	2.32	1.46	1.38	0.57	3.23	3.51
Standard Deviation	0.40	0.50	0.49	0.50	1.14	0.88

Note. Race is coded at 1 = youth of color, 2 = White youth; Substance use type is coded as 1 = cannabis and or alcohol use, 2 = use of other substances with high misuse potential; Polysubstance use is coded as 0 = monosubstance use, 1 = polysubstance use; * *p* < 0.05, ** *p* < 0.01, *** *p* < 0.001.

## Data Availability

The data and the codes can be accessed by emailing the corresponding author.
